# The prevalence of unplanned pregnancy and associated factors in Britain: findings from the third National Survey of Sexual Attitudes and Lifestyles (Natsal-3)

**DOI:** 10.1016/S0140-6736(13)62071-1

**Published:** 2013-11-30

**Authors:** Kaye Wellings, Kyle G Jones, Catherine H Mercer, Clare Tanton, Soazig Clifton, Jessica Datta, Andrew J Copas, Bob Erens, Lorna J Gibson, Wendy Macdowall, Pam Sonnenberg, Andrew Phelps, Anne M Johnson

**Affiliations:** a Department of Social and Environmental Health Research, London School of Hygiene and Tropical Medicine, London, UK; b Research Department of Infection and Population Health, University College London, London, UK; c NatCen Social Research, London, UK

## Abstract

**Background:**

Unplanned pregnancy is a key public health indicator. We describe the prevalence of unplanned pregnancy, and associated factors, in a general population sample in Britain (England, Scotland, and Wales).

**Method:**

We did a probability sample survey, the third National Survey of Sexual Attitudes and Lifestyles (Natsal-3), of 15 162 men and women aged 16–74 years in Britain, including 5686 women of child-bearing age (16–44 years) who were included in the pregnancy analysis, between Sept 6, 2010, and Aug 31, 2012. We describe the planning status of pregnancies with known outcomes in the past year, and report the annual population prevalence of unplanned pregnancy, using a validated, multicriteria, multi-outcome measure (the London Measure of Unplanned Pregnancy). We set the findings in the context of secular trends in reproductive health-related events, and patterns across the life course.

**Findings:**

9·7% of women aged 16–44 years had pregnancies with known outcome in the year before interview, of which 16·2% (95% CI 13·1–19·9) scored as unplanned, 29·0% (25·2–33·2) as ambivalent, and 54·8% (50·3–59·2) as planned, giving an annual prevalence estimate for unplanned pregnancy of 1·5% (1·2–1·9). Pregnancies in women aged 16–19 years were most commonly unplanned (45·2% [30·8–60·5]). However, most unplanned pregnancies were in women aged 20–34 years (62·4% [50·2–73·2]). Factors strongly associated with unplanned pregnancy were first sexual intercourse before 16 years of age (age-adjusted odds ratio 2·85 [95% CI 1·77–4·57], current smoking (2·47 [1·46–4·18]), recent use of drugs other than cannabis (3·41 [1·64–7·11]), and lower educational attainment. Unplanned pregnancy was also associated with lack of sexual competence at first sexual intercourse (1·90 [1·14–3·08]), reporting higher frequency of sex (2·11 [1·25–3·57] for five or more times in the past 4 weeks), receiving sex education mainly from a non-school-based source (1·84 [1·12–3·00]), and current depression (1·96 [1·10–3·47]).

**Interpretation:**

The increasing intervals between first sexual intercourse, cohabitation, and childbearing means that, on average, women in Britain spend about 30 years of their life needing to avert an unplanned pregnancy. Our data offer scope for primary prevention aimed at reducing the rate of unplanned conceptions, and secondary prevention aimed at modification of health behaviours and health disorders in unplanned pregnancy that might be harmful for mother and child.

**Funding:**

Grants from the UK Medical Research Council and the Wellcome Trust, with support from the Economic and Social Research Council and the Department of Health.

## Introduction

A key objective of global public health policy is the reduction of the number of unplanned conceptions.^1^ Available evidence shows that unplanned pregnancies can have a negative effect on women's lives and result in poorer outcomes than those that are planned.^2^, ^3^ Many women with unplanned pregnancies have an abortion, and those who give birth have an increased risk of obstetric complications.^2^, ^3^, ^4^ Women whose pregnancy is unplanned present later for antenatal care,^3^ and are more prone to prenatal and postnatal depression^5^ and relationship breakdown.^3^ Children born of unplanned pregnancies have been shown to have a lower birthweight, have poorer mental and physical health during childhood,^2^, ^3^ and to do less well in cognitive tests.^6^

Estimating the prevalence of unplanned pregnancy and identifying risk factors are crucial to the design of effective preventive interventions. Yet attempts to do so have been beset by methodological challenges.^7^ Despite the fact that becoming pregnant is not always a conscious choice,^8^, ^9^ estimates have often been derived from dichotomous questions about whether pregnancy was planned, or by summing pregnancies reported as unwanted and mistimed.^2^, ^10^ Recognition that a more nuanced method is better suited to capturing the complexity of pregnancy planning status^11^, ^12^, ^13^ has led to the development of more sophisticated approaches, treating planning status as a continuum^14^ and using multi-item measures.^15^, ^16^

In this Article, we present the first estimates of the distribution of pregnancies by planning status, the annual prevalence and factors associated with unplanned pregnancy in Britain (England, Scotland, and Wales) using a psychometrically validated measure. We set these data in the context of demographic and behavioural trends and life-stage transitions. We consider the implications of the findings for public health and clinical practice.

## Methods

### Participants and procedures

The third National Survey of Sexual Attitudes and Lifestyles (Natsal-3) is a stratified probability sample survey of 15 162 men and women aged 16–74 years in Britain (we used data for 5686 women of child-bearing age [16–44 years] for the pregnancy analyses in this paper), interviewed between Sept 6, 2010, and Aug 31, 2012. The response rate was 57·7% and the cooperation rate was 65·8% (of all eligible addresses contacted). We interviewed participants using a combination of computer-assisted face-to-face and self-completion questionnaires. Details of the methods and response calculations are described elsewhere.^17^, ^18^ An anonymised dataset will be deposited with the UK Data Archive, and the complete questionnaire and technical report will be available on the Natsal website on the day of publication.

Survey variables relevant to the questions addressed in this paper included age at interview, and use of contraception at first sexual intercourse; sexual competence at first sexual intercourse (a constructed variable to measure readiness, combining consensuality, autonomy of decision making, timing, and use of effective contraception);^19^ source of sex education; frequency of sex; relationship status; number of children; current smoking and alcohol use; drug use in the past year; and current depression.^20^ Demographic measures include educational attainment; individual socioeconomic status according to the National Statistics Socio-Economic Classification;^21^ and area-level deprivation, using the Index of Multiple Deprivation,^22^ a multidimensional measure combining income, employment, health, education, access to housing and services, crime, and living environment. In the all-age analyses, we defined menopausal status as an age of older than 45 years combined with last menstrual period more than a year ago.^23^

The Natsal-3 study was approved by the Oxfordshire Research Ethics Committee A (Ref: 10/H0604/27). Participants provided oral informed consent for interview.

### Outcomes

To measure the primary outcome, we used the psychometrically validated London Measure of Unplanned Pregnancy (LMUP), developed and validated for use in Natsal-3 from a conceptual model based on qualitative research.^24^, ^25^ Validation in different populations has shown the LMUP to have good psychometric properties.^26^, ^27^ The measure does not assume that women have clearly defined intentions to be pregnant, allows them to express mixed feelings about pregnancy, and can be applied to any pregnancy irrespective of outcome. The self-administered LMUP comprises six questions asking about contraceptive use, timing of motherhood, intention to become pregnant, desire for a baby, discussion with a partner, and pre-conceptual preparations (panel 1). Each item is scored 0–2, the total score ranging from 0–12. Each point increase represents an increase in pregnancy planning and intention, scores of 0–3 being categorised as unplanned, 4–9 as ambivalent, and 10–12 as planned. The LMUP question module was administered to women who were pregnant in the year before interview and, when more than one pregnancy had occurred, in relation to the most recent event.

Panel 1 **London Measure of Unplanned Pregnancy (LMUP) scores**

**Question 1: At the time of conception**

0 Always used contraception
1 Inconsistent use
2 Not using contraception

**Question 2: In terms of becoming a mother**

0 Wrong time
1 OK but not quite right
2 Right time

**Question 3: Just before conception**

0 Did not intend to become pregnant
1 Changing intentions
2 Intended to get pregnant

**Question 4: Just before conception**

0 Did not want a baby
1 Mixed feelings about having a baby
2 Wanted a baby

**Question 5: Before conception**

0 Had never discussed children
1 Discussed but no firm agreement
2 Agreed pregnancy with partner

**Question 6: Before conception**

0 No actions
1 Health preparations (1 action* )
2 Health preparations (≥2 actions*)

### Statistical analysis

We categorised the planning status of pregnancies according to the LMUP. Univariate analysis of data from the subset of women aged 16–44 years who had been pregnant in the previous year was used to describe planning status by age at interview, outcome of pregnancy, relationship status, and number of children. Women whose pregnancies were of unknown outcome at the time of interview were excluded from the analysis to avoid over-representing pregnancies resulting in birth.

We also estimated the proportion of all women aged 16–44 years who experienced an unplanned pregnancy with known outcome in the past year. Logistic regression, adjusting for age, was used to establish factors associated with unplanned pregnancy.

We explored trends in life events related to reproductive health by successive age-group. Survival methods were used to calculate median age and IQR at menarche, first sexual intercourse, first live-in relationship, and first birth. Median ages for all but menarche were plotted by 5-year birth cohort to assess changes over time in the interval between these events.

We used Stata (version 12.1) for all statistical analyses, accounting for stratification, clustering, and weighting of the Natsal-3 dataset.

### Role of the funding source

The sponsors of the study had no role in study design, data collection, data analysis, data interpretation, or writing of the report. The corresponding author had full access to all the data in the study and had final responsibility for the decision to submit for publication.

## Results

15 162 participants (8869 women) aged 16–74 years were recruited to the study with a response rate of 57·7%. Of the 5686 women aged between 16–44 years, 591 (10%) had a pregnancy with known outcome in the year preceding interview and completed the LMUP question module. A further 56 women, whom analysis showed to have a similar demographic profile, did not answer the LMUP module. 44 (7%) of the 591 women who answered the LMUP had more than one pregnancy in the past year.

**Figure 1:**
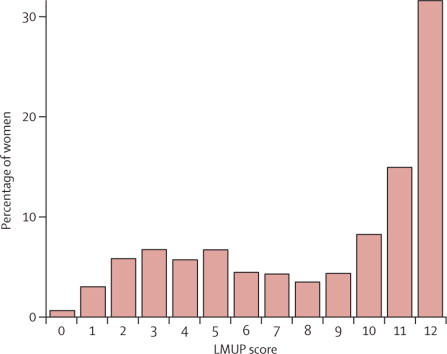
Distribution of London Measure of Unplanned Pregnancy score for women with pregnancy outcome in the year before interview

16·2% of pregnancies with known outcome in the past year scored as unplanned, 29·0% as ambivalent, and 54·8% as planned (figure 1, table 1).

**Table 1:** Planning status of pregnancy by age at interview, outcome, relationship status, and number of children

		**Unplanned (0–3)**		**Ambivalent (4–9)**		**Planned (10–12)**		**LMUP score**	**Denominators (unweighted, weighted)**
		Percentage	95% CI	Percentage	95% CI	Percentage	95% CI	Median (IQR)	
Women reporting pregnancy past year*		16·2%	13·1–19·9	29·0%	25·2–33·2	54·8%	50·3–59·2	10 (5–12)	591, 358
Age at interview† (years)									
	16–19	45·2%	30·8–60·5	43·2%	28·7–59·0	11·6%	5·2–23·8	4 (2–6)	53, 27
	20–24	17·4%	11·9–24·7	42·7%	34·2–51·5	40·0%	31·1–49·6	8 (4–11)	151, 78
	25–29	11·0%	7·3–16·3	26·8%	21·1–33·5	62·2%	54·9–68·9	10 (7–12)	187, 86
	30–34	14·2%	8·4–23·1	18·1%	12·6–25·3	67·7%	58·7–75·5	11 (6–12)	138, 93
	35–44	12·9%	6·2–25·0	25·6%	15·1–40·1	61·4%	47·4–73·8	11 (7–12)	62, 74
Outcome of pregnancy†									
	Full term pregnancy	5·7%	3·7–8·9	28·0%	23·6–32·9	66·3%	61·1–71·0	11 (8–12)	418, 260
	Miscarriage	33·6%	23·2–45·8	31·1%	20·8–43·6	35·3%	25·5–46·6	6 (3–12)	93, 56
	Abortion	57·1%	44·0–69·3	32·5%	22·1–45·0	10·4%	4·4–22·6	2 (2–5)	80, 43
Relationship status at interview†									
	Married or civil partnership	5·2%	2·6–10·4	19·4%	13·8–26·6	75·4%	67·9–81·6	11 (10–12)	228, 171
	Living with a partner	18·2%	12·1–26·6	33·8%	26·9–41·5	48·0%	39·5–56·5	9 (4–12)	179, 114
	Non-cohabiting partnership	39·5%	29·0–51·0	42·8%	32·2–54·1	17·7%	10·7–27·9	4 (2–7)	95, 39
	No partner	38·2%	27·5–50·3	45·8%	34·7–57·4	15·9%	9·6–25·2	5 (3–7)	89, 34
Number of children before most recent pregnancy†									
	0	18·0%	13·5–23·6	24·0%	19·1–29·6	58·1%	51·6–64·2	10 (5–12)	288, 175
	1	7·0%	4·2–11·3	31·1%	23·6–39·8	61·9%	53·2–69·9	11 (7–12)	179, 104
	2+	24·9%	16·8–35·4	38·5%	28·9–49·1	36·6%	27·3–47·0	7 (4–11)	123, 77

LMUP=London Measure of Unplanned Pregnancy.

* Excludes 163 (244 unweighted) women currently pregnant.

† p<0·0001 in a χ^2^ test of association between the factor and the planning status of the pregnancy.

Pregnancy planning status varied substantially with age and pregnancy outcome. Median LMUP score increased with age (table 1). The proportion of pregnancies scored as unplanned was highest among 16–19 year olds and lowest among those aged 25–29 years; the proportion planned was highest among those aged 30–34 years (table 1). Women aged 20–34 years, however, in whom pregnancies more commonly occurred, accounted for nearly two thirds of all unplanned pregnancies (62·4% [95% CI 50·2–73·2]). Ambivalent pregnancies were most prevalent in women aged 16–24 years. Median LMUP score for pregnancies ending in abortion was 2; most were unplanned (table 1). Conversely, most pregnancies ending in birth were planned (table 1).

We saw distinct differences in the planning status of pregnancies by relationship status and number of children. Pregnancies among non-cohabiting women, or those currently without a partner, were more commonly unplanned than those among married or cohabiting women, as were pregnancies among women with no children, or two or more, before the most recent pregnancy, compared with those with one child (table 1).

Overall, 1·5% of women had a pregnancy with known outcome in the year before interview that was defined as unplanned according to the LMUP; the highest prevalence (2·4%) being among 16–19-year-old women (table 2). Pregnancies in 16–19-year-old women accounted for only 7·5% of the total number of pregnancies for all ages, but 21·2% of those that were unplanned (data not shown).

**Table 2:** Prevalence of unplanned pregnancy with outcome in the past year and age-adjusted odds ratios

			**Pregnancy with known outcome in the past year**		Unplanned pregnancy in the past year*		**Adjusted odds ratios for unplanned pregnancy**		**p value**	**Denominators (unweighted, weighted)**
			Percentage	95% CI	Percentage	95% CI	aAOR†	95% CI		
All women aged 16–44 years			9·7%	8·9–10·6	1·5%	1·2–1·9				5686, 3932
Sociodemographic factors										
	Age at interview (years)								0·0361	
		16–19	5·7%	4·3–7·5	2·4%	1·5–3·8	1·00	..	..	975, 506
		20–24	12·2%	10·3–14·4	2·0%	1·3–2·9	0·81	0·44–1·50	..	1137, 685
		25–29	13·2%	11·5–15·1	1·4%	0·9–2·1	0·56	0·30–1·06	..	1383, 687
		30–34	15·8%	13·5–18·4	2·0%	1·2–3·6	0·84	0·40–1·77	..	1016, 644
		35–44	5·5%	4·3–7·0	0·7%	0·3–1·4	0·27	0·11–0·66	..	1175, 1410
	Index of multiple deprivation‡								0·3043	
		1–2 (least deprived)	8·6%	7·4–10·0	1·3%	0·9–2·1	1·00	..	..	1942, 1406
		3	9·6%	7·8–11·7	1·0%	0·5–2·1	0·73	0·32–1·69	..	1117, 790
		4–5 (most deprived)	10·7%	9·5–12·0	1·8%	1·3–2·4	1·28	0·75–2·18	..	2627, 1735
	Occupation code§								0·3822	
		Managerial and professional	10·9%	9·3–12·7	1·5%	0·9–2·4	1·00	..	..	1544, 1217
		Intermediate	9·2%	7·4–11·4	1·0%	0·6–1·8	0·61	0·29–1·32	..	1017, 726
		Semi-routine or routine	12·3%	10·8–14·0	1·9%	1·3–2·7	0·97	0·51–1·84	..	1603, 1043
	Academic qualifications (aged ≥17 years)							..	0·0315	
		Studying for or attained further qualifications	8·4%	7·4–9·5	1·2%	0·9–1·7	1·00	..	..	3028, 2135
		Qualifications typically gained at age 16 years	11·6%	10·1–13·2	1·9%	1·4–2·7	1·98	1·17–3·33	..	1785, 1249
		No qualifications	12·1%	9·3–15·6	1·9%	0·8–4·3	1·88	0·74–4·77	..	458, 296
Health factors										
	Current frequency of binge drinking¶								0·1509	
		Never or rarely	10·3%	9·2–11·4	1·1%	0·8–1·6	1·00	..	..	3391, 2406
		Monthly	6·1%	4·7–7·8	1·4%	0·8–2·3	1·13	0·60–2·13	..	975, 645
		Weekly or daily	6·3%	4·6–8·6	2·3%	1·2–4·2	2·01	1·00–4·07	..	681, 457
	Current smoking status								0·0017	
		Never smoked	8·5%	7·5–9·7	1·1%	0·7–1·6	1·00	..	..	3061, 2189
		Ex-smoker	13·4%	11·2–15·9	1·1%	0·6–2·0	1·29	0·61–2·71	..	932, 685
		Current smoker	9·9%	8·6–11·4	2·6%	1·9–3·5	2·47	1·46–4·18	..	1693, 1058
	Drug use in past year								0·0038	
		No	10·2%	9·3–11·1	1·3%	1·0–1·7	1·00	..	..	4780, 3369
		Yes, cannabis only	6·4%	4·4–9·1	1·4%	0·7–3·1	0·88	0·39–1·99	..	418, 263
		Yes, drugs other than cannabis	11·0%	7·6–15·7	5·2%	2·7–9·7	3·41	1·64–7·11	..	330, 197
	Current depression‖								0·0221	
		No	9·9%	9·0–10·8	1·4%	1·0–1·8	1·00	..	..	4849, 3390
		Yes	10·4%	8·3–13·0	2·7%	1·7–4·5	1·96	1·10–3·47	..	686, 441
Sexual factors										
	Main sex education source								0·0153	
		Lesson at school	7·9%	6·7–9·3	1·0%	0·7–1·6	1·00	..	..	1908, 1301
		Other sources	10·7%	9·7–11·8	1·7%	1·3–2·2	1·84	1·12–3·00	..	3726, 2589
	First sexual intercourse before 16 years							..	<0·0001	
		No	8·8%	7·9–9·7	1·0%	0·7–1·4	1·00	..	..	4075, 2961
		Yes	13·0%	11·3–14·9	3·1%	2·2–4·3	2·85	1·77–4·57	..	1555, 932
	Sexually competent at first sexual intercourse								0·0097	
		Yes	9·6%	8·4–11·0	1·1%	0·8–1·6	1·00	..	..	2467, 1775
		No	11·4%	10·2–12·8	2·1%	1·6–2·8	1·90	1·17–3·08	..	2652, 1806
	Contraception used at first sexual intercourse								0·4761	
		Yes	10·2%	9·3–11·3	1·6%	1·2–2·1	1·00	..	..	4195, 2906
		No	11·7%	9·7–14·1	1·6%	0·9–2·9	1·27	0·66–2·43	..	850, 626
	Opposite-sex partners in past year								0·0208	
		0–1	10·2%	9·3–11·2	1·2%	0·9–1·6	1·00	..	..	4572, 3293
		≥2	7·4%	5·7–9·5	2·9%	1·9–4·5	1·98	1·11–3·53	..	1054, 597
	Frequency of sexual intercourse in past 4 weeks								0·0064	
		0–2 times	8·9%	7·8–10·1	1·1%	0·7–1·6	1·00	..	..	2797, 1909
		3–4 times	10·2%	8·3–12·4	0·9%	0·5–1·7	0·96	0·45–2·01	..	977, 723
		≥5 times	10·7%	9·3–12·3	2·3%	1·7–3·2	2·11	1·25–3·57	..	1859, 1262

* Excludes 48 (27 weighted) women pregnant in the past year for whom the planning of pregnancy could not be established, the denominators reflect this loss.

† Age-adjusted odds ratios (aAORs) are adjusted for participant's age (other than age at interview).

‡ Index of Multiple Deprivation (IMD) is a multi-dimensional measure of area (neighbourhood)-level deprivation based on the participant's postcode: IMD scores for England, Scotland, and Wales were adjusted before being combined and assigned to quintiles, using a method by Payne and Abel.^22^

§ Excludes those in full-time education, those who have never worked, and those who have not worked for more than 10 years.^21^

¶ More than 6 units on one occasion.

‖ Based on the PHQ-2 score (defined by a total score of ≥3 on two screening questions (scored 0–3).^20^

Age-adjusted odds ratios (aAORs) showed unplanned pregnancy to be most strongly associated with occurrence of sexual intercourse before the age of 16 years (table 2). Strong associations were also seen with lack of sexual competence at first sex and with receiving sex education mainly from sources other than school (table 2). Unplanned pregnancy was associated with having sex five or more times in the past 4 weeks and with having more than one heterosexual partner in the past year (table 2). We recorded associations with harder drug use in the past year; current smoking; and current depression (table 2). Lower educational attainment (having no qualifications beyond those associated with minimum school-leaving age) was associated with unplanned pregnancy (table 2). We did further adjustment for education but the changes to the age-adjusted analysis were small and negligible (data not shown).

**Figure 2:**
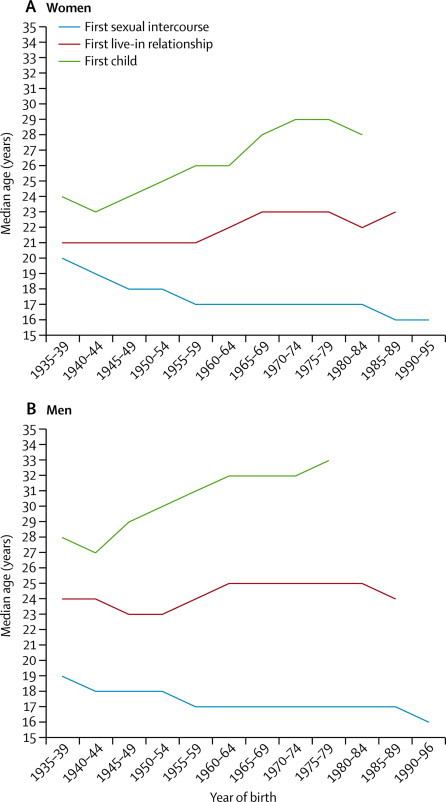
Median age at first sexual intercourse, first live-in relationship, and first child by birth cohort

Analyses by successive age groups (table 3) showed pronounced generational changes in selected single-occurrence events over the six decades represented by the sample. Median age at first sexual intercourse decreased by 3 years for women and 2 years for men, from 19 years for women and 18 years for men aged 65–74 years, to 16 years for both aged under 25 years. Age at first cohabitation and first parenthood, by contrast, increased over the 60 year period, especially in women. Median age at first cohabitation increased by 2 years for women, and by 1 year for men. Median age at first birth increased by 6 years for women (from 23 in those aged 65–74 years to 29 in those aged 25–34 years) and by 5 years for men (aged 28–33 years). Change in the proportion of men and women reporting parenthood before age 20 years was small, but there was a large increase in the proportion of both men and women aged 35 years or older who had not had a child. We detected a gradual increase in the interval between median ages at these life events across 5-year birth cohorts (figure 2).

**Table 3:** Age at menarche (women only), first sexual intercourse, first live-in relationship, and first born child by age at interview

		**Age at interview**							**p value**
		16–24 years	25–34 years	35–44 years	45–54 years	55–64 years	65–74 years	All	
**Women**									
Age at menarche									
	Median age in years (IQR)*	13 (12–14)	13 (12–14)	13 (12–14)	13 (12–14)	13 (12–14)	13 (12–14)	13 (12–14)	..
	Aged <13 years (95% CI)	42·4% (40·1–44·8)	38·8% (36·6–41·1)	37·6% (34·7–40·6)	36·0% (32·8–39·3)	38·3% (35·2–41·6)	35·2% (31·8–38·8)	38·1% (36·9–39·3)	0·0257
	Denominators (unweighted, weighted)	2119, 1194	2467, 1369	1199, 1434	1115, 1432	1024, 1229	866, 927	8790, 7584	..
Age at first sexual intercourse									
	Median age (IQR)*	16 (15–18)	17 (15–18)	17 (16–19)	17 (16–19)	18 (17–20)	19 (17–21)	17 (16–19)	..
	Aged <16 years (95% CI)	29·2% (27·0–31·4)	25·1% (23·3–27·1)	18·1% (15·8–20·6)	14·3% (12·2–16·7)	9·9% (8·1–12·1)	4·0% (2·8–5·9)	17·4% (16·5–18·3)	<0·0001
	Denominators (unweighted, weighted)	2110, 1190	2469, 1370	1196, 1428	1106, 1418	1015, 1216	850, 911	8746, 7533	..
	Lacking sexual competence (95% CI)†	51·9% (49·3–54·6)	48·7% (46·5–50·9)	51·3% (48·3–54·4)	54·1% (50·8–57·3)	56·7% (53·4–59·9)	57·4% (53·6–61·0)	53·1% (51·8–54·4)	0·0005
	Denominators (unweighted, weighted)	1679, 936	2381, 1316	1175, 1402	1075, 1388	988, 1189	822, 880	8120, 7112	..
Age at first live-in relationship									
	Median age (IQR)*	23 (20–‡)	23 (20–26)	23 (20–27)	22 (19–25)	21 (19–24)	21 (19–23)	22 (20–25)	..
	Aged <20 years (95% CI)	23·7%‡‡ (21·1–26·5)	22·6% (20·9–24·5)	20·9% (18·3–23·7)	25·5% (22·7–28·6)	28·9% (26·0–32·0)	29·3% (26·1–32·6)	24·9% (23·8–26·1)	<0·0001
	Denominators (unweighted, weighted)	2092, 1183	2413, 1341	1183, 1417	1076, 1385	1002, 1203	851, 908	8617, 7436	..
Age at first-born child									
	Median age (IQR)*	§ (22–‡)	29 (23–‡)	28 (23–35)	26 (22–32)	25 (21–31)	23 (21–27)	26 (22–33)	..
	Aged <20 years (95% CI)	14·0%¶ (12·1–16·2)	12·9% (11·7–14·3)	10·5% (8·6–12·7)	12·1% (10·1–14·3)	14·8% (12·6–17·2)	14·4% (12·0–17·1)	12·9% (12·1–13·7)	0·0324
	No child before age 35 years	NA	NA	25·4% (22·8–28·2)	19·4% (17·2–21·8)	19·4% (16·9–22·1)	13·8% (11·5–16·6)	20·2% (18·9–21·5)	<0·0001
	Denominators (unweighted, weighted)	2124, 1198	2428, 1345	1179, 1412	1089, 1400	993, 1187	818, 871	8631, 7414	..
**Men**									
Age at first sexual intercourse									
	Median age (IQR)*	16 (15–18)	17 (15–19)	17 (15–19)	17 (15–18)	18 (16–19)	18 (16–21)	17 (16–19)	..
	Aged <16 years (95% CI)	30·9% (28·5–33·5)	25·5% (23·1–28·1)	26·6% (23·4–30·1)	26·7% (23·4–30·3)	17·3% (14·6–20·5)	15·4% (12·6–18·6)	24·4% (23·1–25·7)	<0·0001
	Denominators (unweighted, weighted)	1712, 1228	1508, 1362	792, 1396	780, 1389	758, 1175	657, 846	6207, 7296	..
	Lacking sexual competence (95% CI)†	43·8% (40·6–47·0)	46·6% (43·7–49·5)	53·4% (49·5–57·3)	56·3% (52·2–60·2)	61·1% (57·2–64·9)	67·4% (63·3–71·1)	54·2% (52·7–55·7)	<0·0001
	Denominators (unweighted, weighted)	1323, 970	1424, 1279	773, 1364	744, 1332	721, 1130	625, 809	5610, 6885	..
Age at first live-in relationship									
	Median age (IQR)*	§ (22–‡)	25 (22–29)	25 (22–30)	25 (22–29)	23 (21–28)	24 (22–26)	24 (22–29)	..
	Aged <20 years (95% CI)	11·4%‖ (9·4–13·7)	10·1% (8·6–11·9)	9·9% (7·9–12·3)	9·8% (7·8–12·2)	12·0% (9·7–14·7)	8·3% (6·2–11·0)	10·2% (9·4–11·2)	0·3354
	Denominators (unweighted, weighted)	1712, 1227	1489, 1343	777, 1372	756, 1354	741, 1155	637, 828	6112, 7280	..
Age at first-born child									
	Median age (IQR)*	NA**	33 (27–‡)	32 (27–42)	31 (26–‡)	30 (25–‡)	28 (24–36)	31 (26–59)	..
	Aged <20 years (95% CI)	3·0%†† (2·0–4·4)	3·1% (2·3–4·2)	3·7% (2·4–5·7)	3·3% (2·2–4·9)	4·2% (2·9–6·0)	2·6% (1·5–4·6)	3·4% (2·9–4·0)	0·6907
	No child before age 35 years	NA	NA	40·6% (37·0–44·3)	40·4% (36·7–44·1)	38·4% (34·6–42·4)	26·4% (22·9–30·2)	37·6% (35·7–39·6)	<0·0001
	Denominators (unweighted, weighted)	1710, 1226	1496, 1349	789, 1393	764, 1355	722, 1122	598, 775	6079, 7220	..

* Medians and centiles were calculated with survival methods.

† Excludes participants who have not had sexual intercourse yet.

‡ Value for 75th percentile was censored (ie, <75% of the age group had the event).

‡‡ Figure excludes 965 (502 weighted) women younger than 20 years.

§ Value for median was censored (ie, <50% of the age group had the event).

¶ Figure excludes 978 (509 weighted) women younger than 20 years.

‖ Figure excludes 851 (529 weighted) men younger than 20 years.

** Values for 25th percentile, median, and 75th percentile were censored.

†† Figure excludes 949 (527 weighted) men younger than 20 years.

**Figure 3:**
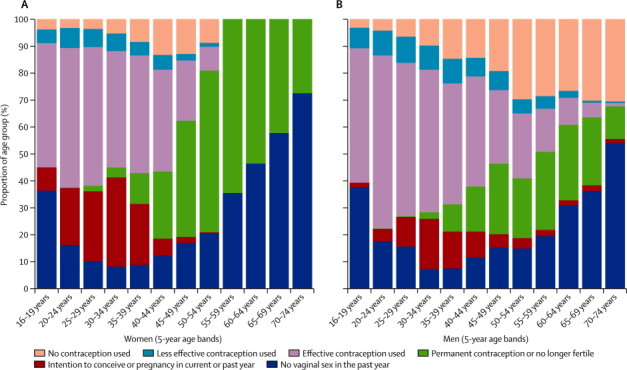
Behaviours relating to pregnancy risk in the year before interview, by age-group

Figure 3 shows changes in pregnancy prevalence, intention, and contraceptive protection through the life course. In the youngest age group, 16–19 years, sexual inactivity is more common and being pregnant or planning to conceive is rare, but although use of effective contraception by those who are sexually active is high, a few use less effective methods including methods used after unprotected sex. Through their 20s, many more men and women are sexually active and pregnancy experience and intention become more common but use of contraception remains high and effective methods are more commonly used than less effective ones. Intention to conceive peaks in the early 30s and remains high until after age 40 years, when the risk of conception is reduced by use of permanent contraception, post-menopausal status, and sexual inactivity.

## Discussion

We know of no other study that has provided population prevalence estimates of unplanned pregnancy in Britain using a validated measure. Roughly one in six pregnancies occurring in the year before interview (done between September, 2010, and August, 2012) were unplanned, between a quarter and a third were ambivalent, and over half were planned. If we apply our estimates to national data, 159 656 (16·2%) of the 985 528 pregnancies recorded in Britain in 2011 would be categorised as unplanned, 285 803 (29·0%) as ambivalent, and as 540 069 (54·8%) as planned.

Overall, nearly one in 60 women had an unplanned pregnancy in the previous year. We recorded strong associations between unplanned pregnancy and health-related factors (ie, current smoking, drug use, and depression), lower educational attainment, and aspects of sexual behaviour (ie, early initiation of sexual activity, lack of sexual competence at first sexual intercourse, receipt of sex education from sources other than school, higher frequency of recent sex, and reporting more than one heterosexual partner in the past year).

Our data show marked generational changes, in little more than half a century, in the timing of events related to reproductive health, notably a progressive decrease in age at first sexual intercourse and an increase in the age at first cohabitation and becoming a parent. As a consequence of the increasing interval between these events, and the trend towards smaller families, a heterosexually active woman in Britain might now spend some 30 years of her life needing to avert unplanned pregnancy.

The strength of this study is that it is population-wide, produces estimates of planning status for pregnancy resulting in abortions, miscarriages, and births, and uses a dedicated, validated, multi-dimensional measure of unplanned pregnancy. A weakness is that it is cross-sectional, and so chronology cannot always be determined, nor can causality be inferred. Contraception practice, for example, was recorded for the past year and might have preceded or followed conception.

A further limitation is that we relied on self-reported data, which can be subject to recall and desirability bias. Perceptions of the circumstances of pregnancy may be recast over time.^28^ Restriction of the period during which women were required to reflect on these to 1 year would have minimised difficulty recalling these circumstances, and specific questions included in the measure relating to activities such as pre-pregnancy care and contraceptive use are less likely to be recast over time, but prospective studies remain the ideal design to reduce after-the-fact rationalisation.^16^

Our prevalence estimates are based on pregnancies with known outcome in the year before interview, some of which would have been conceived in the previous year. Because this number is balanced by an equal number of pregnancies conceived in the current year and ongoing at the time of interview, which were treated in the analysis as if not pregnant, we were able to produce an estimate of the annual prevalence of unplanned conception. However, the official UK figures in 2011 for the abortion ratio—the number of abortions per 1000 livebirths—was 251 (compared with 165 in the Natsal-3 sample).^29^ Adjustment of our estimates to take account of this discrepancy would put the overall proportion of pregnancies unplanned at 18·9%.

Comparisons of our estimates of unplanned pregnancy with others are made difficult by differences in measurement, differences in the populations under study, and because estimates are often applied to only births and not abortions. Our estimates of the proportion of births that are unplanned, ambivalent, or planned are similar to those from studies that used the LMUP in clinical samples in Scotland.^31^ Our estimate of the proportion of abortions that are unplanned is lower than that seen in the Scottish studies in which the LMUP questions were asked of women at the time of clinical consultation^30^, ^32^—a time when there might be a need for greater conviction to bolster the decision taken.

Estimates from other high-income countries are higher. In France, a third of pregnancies are estimated to be unplanned,^33^ two in five in Spain,^34^ almost half in Japan,^35^ and between a third and a half in the USA.^36^, ^37^ In these studies, estimates were derived either from a single question asking about pregnancy intention, or by combining pregnancies reported as unwanted and mistimed. In US studies in which the LMUP has been used, the proportion of pregnancies estimated as unplanned was 30% in California^26^ and 28% in Pennsylvania.^16^ Thus although our data do not lend support to the claim that the proportion in Britain is only a third of that in the USA,^38^ they do suggest that it may be lower. The lower proportion of unplanned pregnancies in Britain might be because contraceptive services and abortion are readily available and financially subsidised in the UK—an interpretation supported by increasing evidence of the effects of provision of free contraception in lowering unplanned pregnancy rates.^39^, ^40^

Our findings have practical public health and clinical importance. The strong associations seen in our study between unplanned pregnancy and age, relationship status, education, potentially harmful health behaviours, and depression are consistent with those from other studies (panel 2),^4^, ^35^, ^36^, ^41^ and offer much scope for public health and clinical intervention. Information about the profile of women most at risk of unplanned pregnancy will be of value in targeting preventive efforts. The term unplanned is often uncritically applied to pregnancies in younger women and those ending in abortion.^7^ Our data substantiate the idea that interventions to prevent unintended pregnancy should target young single women. Nevertheless, half of unplanned pregnancies in our sample were in women aged 25–34 years, and the public health effect in older age groups of women also needs to be recognised and appropriate services provided. Similarly, our finding that four in ten pregnancies that were terminated were planned or ambivalent cautions against equating abortion with unplanned pregnancy.

Panel 2 **Research in context**

**Systematic review**

The investigative scope of the Natsal-3 surveys has extended in successive studies and there is now a greater focus on reproductive health. For the first time, in Natsal-3, we measured unplanned pregnancy. We provide the first prevalence estimates of unplanned pregnancy in Britain since those of Fleissig in 1989,^46^ and the first ever using a multi-component, psychometrically validated measure, the London Measure of Unplanned Pregnancy (LMUP). The LMUP, based on a conceptual model derived from qualitative interviews with women in Britain, was developed specifically for Natsal-3 and has now been validated in several studies.^26^, ^27^, ^30^, ^31^, ^32^

**Interpretation**

Comparisons of our estimates with those from other high-income countries suggest that the prevalence of unplanned pregnancy is lower in Britain. The factors we showed to be associated with unplanned pregnancy: age, relationship status, lower educational level, sex education from non-school sources, and sexual and health risk behaviours, are similar to those seen in other studies and offer scope for primary and secondary prevention. The LMUP might be useful in clinical settings as well as research settings to identify women at risk of adverse outcomes of pregnancy. Our findings will also be useful in guiding strategies to prevent unplanned conception, and also those aimed at mitigating the adverse outcomes of unplanned pregnancy for mother and child.

In the context of primary prevention strategies—those aimed at reducing the number of pregnancies that are unplanned—our finding that unplanned pregnancy was more strongly associated with educational attainment than with socioeconomic status has particular relevance. That the most effective contraception is education is well known. Improvement in women's lives, education, and employment options are probably the strongest motivators to avoid unintended pregnancy. The potential of early sexual experiences in predicting, and school-based sex education in preventing, unplanned pregnancy emphasises the importance of effective sexual and reproductive health care from an early age.

The higher prevalence of unplanned pregnancy in nulliparous women and those with two or more children, compared with those with one, suggests the need for fertility intervention to shift their focus on excess births in the middle to unexpected pregnancies at the beginning or the end of the family-building cycle.^9^ In countries with well organised and free-of-charge contraceptive provision, appropriate interventions will probably be focused less on unmet need and more on reduction of contraceptive failure, through health education, skill building, counselling, and advocacy of long-acting methods of contraception.^42^

Our findings also lend support to pleas for greater attention to secondary preventive strategies—those aimed at mitigating adverse outcomes of unplanned pregnancy.^43^ In this context, a measure that takes account of both negative and positive feelings about a pregnancy has value in clinical settings as well as in epidemiological research. We used the term ambivalence to represent a midway position between planned and unplanned, rather than an affect expressed by women themselves. Yet there is increasing recognition that many women have mixed feelings about pregnancy.^44^ Intervening at a time when attitudes towards a pregnancy might be labile and subject to revision offers scope for helping women to mitigate the adverse outcomes of unplanned pregnancy.^45^ The association between unplanned pregnancy and poorer mental health prompts practitioners to consider the possibility and consequences of depression in pregnancy. The strong association with potentially unhealthy behaviours such as smoking and drug use emphasises the need to help women and their partners to modify aspects of lifestyle that can harm their own health and wellbeing, and that of their child.

## Supplementary Material

Supplementary video Results of over 45 000 respondents, recording the dramatic changes in our sexual lifestyles over the past 60 years.

## Footnotes

* Health preparations included the following actions: taking folic acid supplements, stopping or reducing smoking, stopping or reducing alcohol consumption, healthy eating, and seeking medical advice before conception.
